# Carbapenemase-Producing *Enterobacteriaceae* Colonization and the Risk of Carbapenemase-Producing *Enterobacteriaceae* Bacteremia in Hematopoietic Stem Cell Transplant Recipients

**DOI:** 10.1093/ofid/ofaf516

**Published:** 2025-09-02

**Authors:** Sung-Woon Kang, So Yun Lim, Euijin Chang, Jiwon Jung, Yong Pil Chong, Hyunkyung Park, Han-Seung Park, Yunsuk Choi, Jung-Hee Lee, Je-Hwan Lee, Sung-Han Kim

**Affiliations:** Department of Infectious Diseases, Asan Medical Center, University of Ulsan College of Medicine, Seoul, Republic of Korea; Department for Infection Control, Armed Forces Daejeon Hospital, Daejeon, Republic of Korea; Department of Infectious Diseases, Asan Medical Center, University of Ulsan College of Medicine, Seoul, Republic of Korea; Department of Infectious Diseases, Asan Medical Center, University of Ulsan College of Medicine, Seoul, Republic of Korea; Department of Infectious Diseases, Asan Medical Center, University of Ulsan College of Medicine, Seoul, Republic of Korea; Department of Infectious Diseases, Asan Medical Center, University of Ulsan College of Medicine, Seoul, Republic of Korea; Department of Hematology, Asan Medical Center, University of Ulsan College of Medicine, Seoul, Republic of Korea; Department of Hematology, Asan Medical Center, University of Ulsan College of Medicine, Seoul, Republic of Korea; Department of Hematology, Asan Medical Center, University of Ulsan College of Medicine, Seoul, Republic of Korea; Department of Hematology, Asan Medical Center, University of Ulsan College of Medicine, Seoul, Republic of Korea; Department of Hematology, Asan Medical Center, University of Ulsan College of Medicine, Seoul, Republic of Korea; Department of Infectious Diseases, Asan Medical Center, University of Ulsan College of Medicine, Seoul, Republic of Korea

**Keywords:** carbapenemase-producing *Enterobacteriaceae*, hematopoietic stem cell transplantation

## Abstract

**Background:**

Carbapenemase-producing *Enterobacteriaceae* (CPE) are globally concerning pathogens due to limited therapeutic options. Despite the increasing incidence of CPE infections, evidence supporting effective empirical treatments for individuals undergoing hematopoietic stem cell transplantation (HSCT) remains limited.

**Methods:**

From January 2019 to December 2023, individuals undergoing HSCT screened for CPE colonization via perianal swabs upon admission before HSCT were retrospectively analyzed. Culture-based identification and carbapenemase-specific polymerase chain reaction were performed. The occurrence of *Enterobacteriaceae* bacteremia within 100 days post-HSCT was monitored. Propensity-score (PS) matching and competing risk analyses were used to evaluate the relationship between CPE colonization and bacteremia risk.

**Results:**

Among 649 patients undergoing HSCT, 70 (11%) were colonized with CPE. *Enterobacteriaceae* bacteremia occurred in 20 (29%) CPE-colonized and 56 (10%) noncolonized individuals (*P* < .001). Among these cases, 17/20 (85%) in the colonized group and 12/56 (21%) in the noncolonized group were caused by CPE (*P* < .001). After 1:2 PS matching, these rates remained consistent (85% vs 22%, *P* = .004). All CPE isolates recovered from blood were identical in species and carbapenemase type to those detected in pre-HSCT swabs. Competing risk analyses showed that pre-HSCT CPE colonization was significantly associated with CPE bacteremia (subdistribution hazard ratio [sHR] 13.1, 95% confidence interval [CI] 6.27–27.3, *P* < .001; after matching: sHR 19.1, 95% CI 4.42–82.20, *P* < .001).

**Conclusions:**

Pre-HSCT CPE colonization increases *Enterobacteriaceae* bacteremia risk. Routine screening and empirical CPE-directed therapy are essential to improving clinical outcomes.

The global dissemination of carbapenemase-producing *Enterobacteriaceae* (CPE) and the resultant surge in CPE infections have emerged as significant global health concerns [1]. Among immunocompromised individuals, particularly those with hematologic malignancies, CPE infections are associated with increased mortality due to limited therapeutic options [2–4]. Identifying risk factors for CPE infections in this vulnerable population is therefore crucial.

Guidelines from the Infectious Diseases Society of America (IDSA) and the European Conference on Infections in Leukemia (ECIL) recommend empirical CPE-targeted therapy for neutropenic infections suspected to involve CPE; however, supporting evidence remains limited [2, 5]. Additionally, the indiscriminate use of novel antimicrobials effective against CPE might contribute to resistance, further reducing already limited therapeutic options [5]. Consequently, high-quality evidence investigating the epidemiology and risk factors for CPE infections in patients with neutropenia is urgently required.

Several studies have investigated the risk factors for CPE bacteremia in individuals undergoing hematopoietic stem cell transplantation (HSCT) [6–9]. However, these studies often have limited sample sizes and heterogeneity in underlying diseases, resulting in weak overall evidence [10]. To address these limitations, a study that appropriately adjusts for patients' underlying conditions and accounts for early mortality was designed. At Asan Medical Center, CPE screening was initially performed at admission on an as-needed basis during outbreaks in specific wards, including HSCT units, from 2019 to June 2020. Since June 2020, hospital-wide screening at admission has been implemented. This study includes on patients admitted for HSCT who underwent CPE screening at admission, to evaluate the association between positive screening results and *Enterobacteriaceae* bacteremia during the posttransplant period.

## METHODS

### Study Population, Study Design, and Ethical Approval

From 1 January 2019, to 31 December 2023, adult (≥18 years old) individuals undergoing HSCT at Asan Medical Center, a 2700-bed teaching hospital in Seoul, South Korea, who underwent perianal swab-based CPE screening at admission, were retrospectively reviewed. Carbapenemase-producing *Enterobacteriaceae*–specific cultures and real-time polymerase chain reaction (PCR) for carbapenemase (Xpert Carba-R assay) were performed on the collected swabs. Patients colonized with CPE were isolated in single-bed rooms, and contact precautions were implemented. To evaluate the risk of subsequent CPE bacteremia, the first episode of *Enterobacteriaceae* bacteremia occurring within 100 days of HSCT was identified. All patients received global prophylaxis with trimethoprim-sulfamethoxazole, from the time of engraftment to 6 months posttransplantation. Quinolone prophylaxis was recommended from the time of cell infusion until neutrophil recovery (absolute neutrophil count ≥ 500 cells/μL), unless patients had already received broad-spectrum antibiotics for other indications, such as febrile neutropenia. However, quinolone prophylaxis was discontinued after February 2024 due to concerns about the emergence of resistant pathogens, such as CPE. In cases of febrile neutropenia, initial empiric therapy with cefazolin and ceftazidime was administered. If fever persisted for more than 3 days despite empirical therapy, escalation to cefepime or piperacillin/tazobactam was recommended, unless hemodynamic instability was present. If the patient was hemodynamically unstable, empirical therapy with meropenem, vancomycin (or teicoplanin), amikacin, and liposomal amphotericin B (or caspofungin) was initiated. In hemodynamically unstable patients colonized with CPE, empiric therapy with amikacin or ceftazidime/avibactam was considered. If there was no evidence of CPE bacteremia within 2 days, de-escalation to cefepime or piperacillin/tazobactam was recommended. The testing, prophylaxis, and empiric treatment protocols during HSCT are summarized in Supplementary Table 1. Data on patient demographics, Charlson's comorbidity index, malignancy type, and transplant characteristics were analyzed. This study was approved by the institutional review board of Asan Medical Center (IRB-2025-0075).

### Definitions

Carbapenemase-producing *Enterobacteriaceae* was defined as carbapenem-resistant *Enterobacteriaceae* (CRE) producing any carbapenemase type, including *Klebsiella-producing* carbapenemase (KPC), New Delhi metallo-beta-lactamase (NDM), imipenemase metallo-beta-lactamase, Verona integrin-encoded metallo-beta-lactamase (VIM), and oxacillinase-48-like beta-lactamase (OXA-48-like) enzymes [3]. Carbapenemase-producing *Enterobacteriaceae* colonization was defined as a positive carbapenemase-specific PCR result from a perianal swab collected at admission. The Centers for Disease Control and Prevention criteria were used to evaluate the suspected source of bacteremia [11]. Febrile neutropenia was defined as a neutrophil count of ≤500 cells/mm^3^ accompanied by fever (≥38°C). Neutrophil engraftment was defined as 3 consecutive days with a neutrophil count of ≥500 cells/mm^3^. Prior hospitalization, prior antibiotic exposure, and prior CPE infection were defined as those occurring within 6 months before transplantation.

### Microbiological Data

Perianal swabs collected for CPE screening were cultured without broth enrichment step using ChromID CARBA agar (bioMérieux, France), with species identification performed using matrix-assisted laser desorption ionization-time of flight mass spectrometry (Bruker, Bremen, Germany). Concurrently, the Xpert Carba-R assay (Cepheid, Sunnyvale, CA, USA) was conducted on collected swabs. Species identification and antimicrobial susceptibility testing for blood isolates were performed using the MicroScan WalkAway 96 plus and Neg Combo Panel Type 72 (Beckman Coulter, Brea, CA, USA), following Clinical and Laboratory Standards Institute guidelines. All CRE blood isolates underwent the modified Hodge test and a carbapenemase inhibition assay using phenylboronic acid and ethylenediaminetetraacetic acid to assess carbapenemase activity [12]. The presence of carbapenemase genes in blood isolates was further confirmed using the Xpert Carba-R assay. Organisms that grew on the ChromID CARBA agar but were negative on the Carba-R assay were classified as CRE.

### Statistical Analysis

Categorical variables were analyzed using the chi-square or Fisher's exact test, while continuous variables were assessed using Wilcoxon rank-sum test. A *P*-value of <.05 in 2-tailed tests was considered statistically significant. In this study, patients could experience 3 types of events within a competing-risk framework: CPE bacteremia as the first *Enterobacteriaceae* bacteremia, non-CPE bacteremia, or death before developing any *Enterobacteriaceae* bacteremia. To address this, a competing risk analysis was planned using the Fine–Gray model. Multivariate survival analysis with backward regression was conducted as a sensitivity analysis, treating death before *Enterobacteriaceae* bacteremia and non-CPE bacteremia as right-censoring events.

To adjust for potential confounders, we performed 1:2 nearest-neighbor propensity-score (PS) matching between CPE-colonized and noncolonized patients without replacement. The PS was generated using logistic regression based on baseline demographics, underlying diseases, Charlson's comorbidity index, hematologic malignancy type, and transplant characteristics. A standardized mean difference of <0.1 was considered a well-balanced match. Statistical analyses were performed using R software.

## RESULTS

### Baseline Characteristics

**Figure 1.**
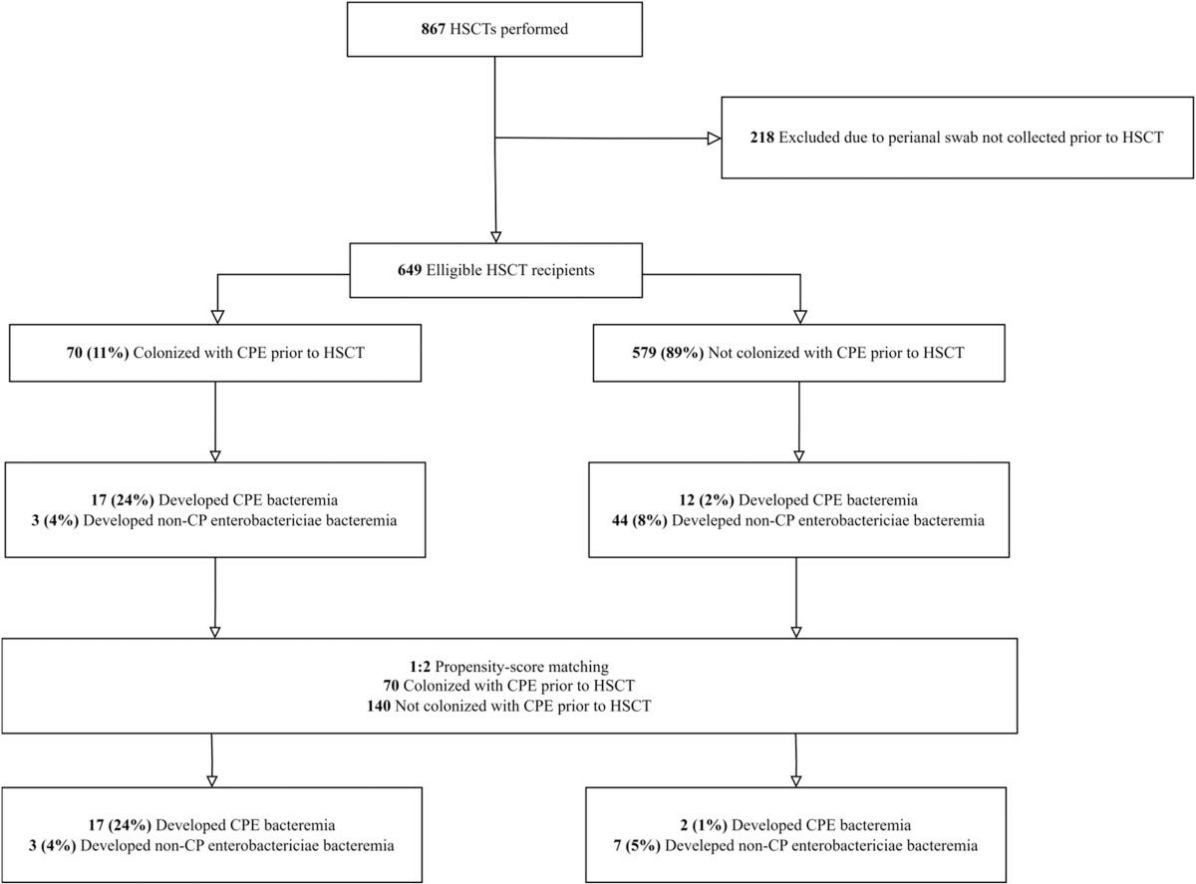
Flow diagram of patients included in the study and their risk of CPE bacteremia. CPE, carbapenemase-producing *Enterobacteriaceae*; HSCT, hematopoietic stem cell transplantation.

During the study period, 867 patients underwent HSCT, of whom 649 (75%) were screened for CPE colonization (Figure 1). Among these, 70 recipients tested positive for CPE colonization, while remaining 579 recipients were not colonized (Table 1). Compared with noncolonized recipients, CPE-colonized recipients were younger and more likely to have hypertension (29% vs 18%, *P* = .05), acute myeloid leukemia (46% vs 33%), and acute lymphoblastic leukemia (33% vs 14%). They were also more likely to have received allogeneic HSCT (87% vs 75%). Additionally, CPE-colonized recipients were less likely to receive fluoroquinolone prophylaxis (21% vs 40%, *P* = .003) and had a higher 90-day mortality rate (14% vs 5%, *P* = .005).

**Table 1.** Baseline Characteristics and Outcome Variables of the Study Population based on the Colonization Status

Variable	Colonized With CPE (N = 70)	Not Colonized With CPE (N = 579)	Total (N = 649)	*P*-Value
Age (y), mean ± SD	49.3 ± 14.1	52.5 ± 13.3	52.2 ± 13.4	.06
Male	45 (64)	316 (55)	361 (56)	.16
Underlying disease/condition				
Hypertension	20 (29)	104 (18)	124 (19)	.05
Diabetes mellitus	11 (16)	90 (16)	101 (16)	>.99
Chronic kidney disease	4 (6)	19 (3)	23 (4)	.49
Liver cirrhosis	0	4 (1)	4 (1)	.99
Solid organ transplant	1 (1)	4 (1)	5 (1)	.40
Solid cancer	1 (1)	18 (3)	19 (3)	.68
Rheumatologic disease	4 (6)	14 (2)	18 (3)	.23
Charlson's comorbidity index	3.4 ± 1.6	3.6 ± 1.5	3.6 ± 1.5	.28
Underlying malignancy				<.001
AML	32 (46)	189 (33)	221 (34)	
ALL	23 (33)	82 (14)	105 (16)	
Multiple myeloma	6 (9)	110 (19)	116 (17)	
MDS	2 (3)	108 (19)	110 (17)	
CML	1 (1)	9 (2)	10 (2)	
Non-Hodgkin's lymphoma	5 (7)	51 (9)	56 (9)	
Hodgkin's lymphoma	1 (1)	4 (1)	5 (1)	
Others	1 (1)	35 (6)	36 (6)	
Type of transplant				.04
Allogeneic	61 (87)	436 (75)	497 (77)	
Autologous	9 (13)	143 (25)	152 (23)	
Source of donor				.22
Matched, related	14 (20)	100 (17)	114 (18)	
Matched, unrelated	24 (34)	160 (28)	184 (28)	
Mismatched, related	22 (31)	160 (28)	182 (28)	
Mismatched, unrelated	1 (1)	16 (3)	17 (3)	
Conditioning regimen				.02
Myeloablative	70 (100)	529 (91)	599 (92)	
Reduced-intensity or nonmyeloablative	0	50 (9)	50 (8)	
Prior hospitalization	66 (94)	398 (69)	464 (72)	<.001
Prior CPE infection	2 (3)^a^	3 (1)^b^	5 (1)	.16
Antibiotics exposure	69 (99)	477 (82)	546 (84)	.001
Gram-negative coverage	69 (99)	468 (81)	537 (83)	<.001
Quinolone prophylaxis	15 (21)	233 (40)	248 (38)	.003
Non-*Enterobacteriaceae* bacteremia	11 (16)^c^	67 (12)^d^	78 (12)	.42
Non-CPE bacteremia	3 (4)	44 (8)	47 (7)	.46
Duration from perianal swab to transplantation, mean days ± SD^e^	8.0 ± 6.4	7.2 ± 2.6	7.3 ± 3.2	.94
Duration to neutrophil engraftment, mean days ± SD^f^	12.3 ± 9.0	10.7 ± 8.6	10.9 ± 8.7	.14
30-d mortality after HSCT	1 (1)	13 (2)	14 (2)	.99
90-d mortality after HSCT	10 (14)	29 (5)	39 (6)	.005

Data are presented as number (%) unless otherwise indicated.

Abbreviations: CPE, carbapenemase-producing *Enterobacteriaceae*; SD, standard deviation; AML, acute myeloid leukemia; ALL, acute lymphoblastic leukemia; MDS, myelodysplastic syndrome; CML, chronic myeloid leukemia; CoNS, coagulase-negative *Staphylococcus*.

^a^Two patients with CPE bacteremia.

^b^One patient with CPE pneumonia and two patients with CPE bacteremia.

^c^Six patients with Enterococcus bacteremia, two patients with CoNS bacteremia, two patients with *Pseudomonas aeruginosa* bacteremia, and one patient with *Achromobacter xylosoxidans* bacteremia.

^d^Twenty-two patients with CoNS bacteremia, 15 patients with Enterococcus bacteremia, 12 patients with Corynebacterium bacteremia, 6 patients with Streptococcus bacteremia, 5 patients with Capnocytophaga bacteremia, 2 patients with *Bacillus cereus* bacteremia, 2 patients with *P. aeruginosa* bacteremia, 1 patient with Fusobacterium bacteremia, 1 patient with *Clostridium tertium* bacteremia, and 1 patient with *Sphingomonas paucimobilis* bacteremia.

^e^Days from surveillance rectal swab to HSCT.

^f^Neutrophil engraftment was defined as days from cell infusion to the resolution of neutropenia (3 consecutive days with a neutrophil count of ≥500 cells/mm^3^).

### Risk and Characteristics of Carbapenemase-Producing *Enterobacteriaceae* Bacteremia

**Figure 2.**
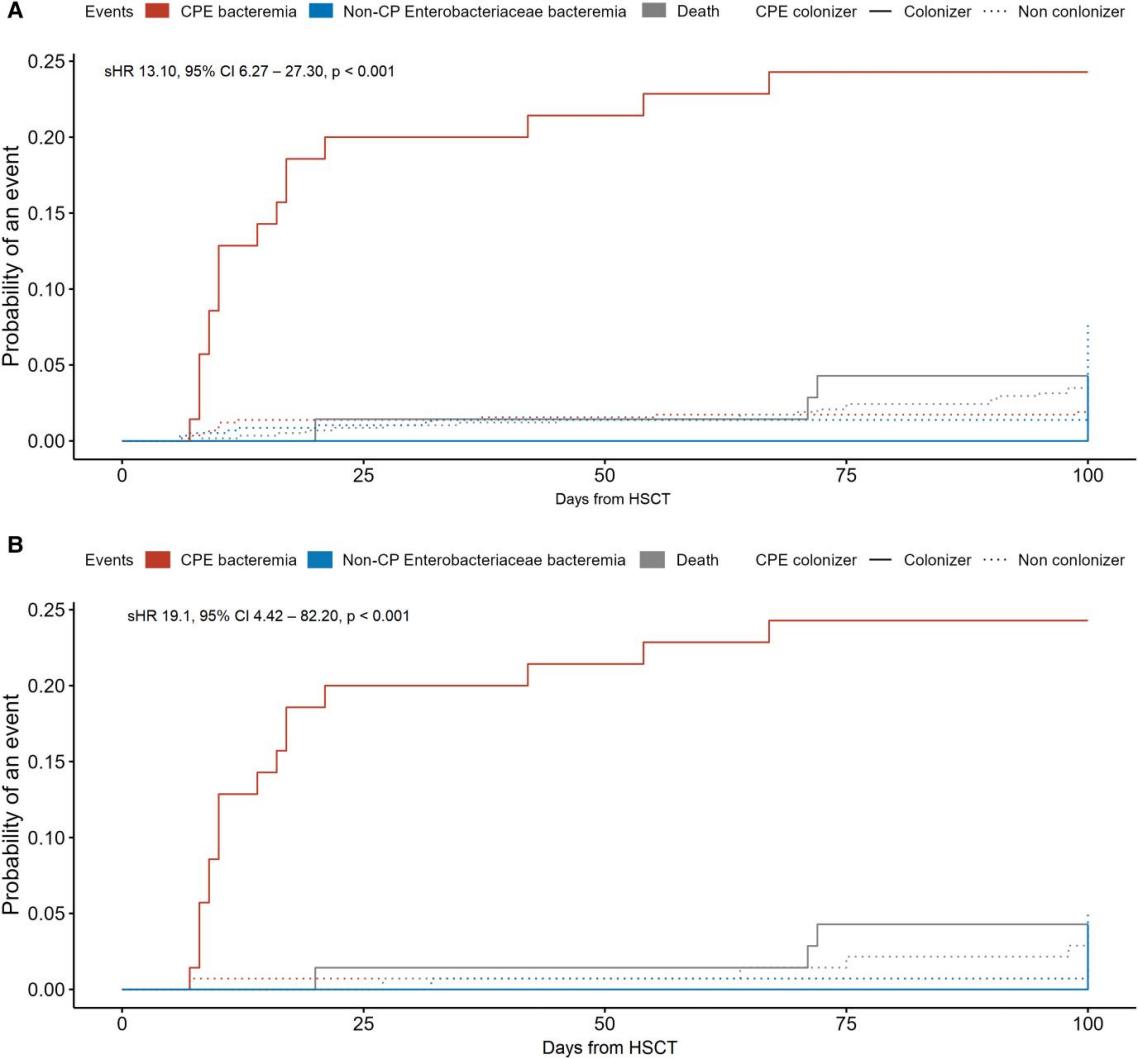
Cumulative incidence plot of CPE bacteremia based on the colonization status. The red line represents CPE bacteremia, the blue line represents non-CPE bacteremia, and the gray line indicates death before the occurrence of *Enterobacteriaceae* bacteremia. Solid lines denote CPE-colonizers, while dotted lines represent noncolonizers. *A*, Risk of CPE bacteremia in individuals with and without CPE colonization. *B*, Risk of CPE bacteremia in individuals with and without CPE colonization, after propensity-score matching. CPE, carbapenemase-producing *Enterobacteriaceae*; sHR, subdistribution hazard ratio; CI, confidence interval; HSCT, hematopoietic stem cell transplantation.

Carbapenemase-producing *Enterobacteriaceae* bacteremia occurred more frequently in the CPE-colonized group compared with the noncolonized group (17 patients [24%] vs 12 patients [2%], *P* < .001; Figure 1). An additional case of KPC-producing *Klebsiella pneumoniae* bacteremia occurred in the noncolonized group after an initial non-CP *K. pneumoniae* bacteremia episode, but it was excluded from the analysis as a second event. Additionally, *Enterobacteriaceae* bacteremia was more common in CPE-colonized recipients than in noncolonized recipients (20 patients [27%] vs 56 patients [10%], *P* < .001). The time from HSCT to CPE bacteremia did not differ significantly between the 2 groups (median 10 days, interquartile range [IQR] 9.0–17.0 vs median 10 days, IQR 8.5–46.0, *P* = .96). Competing-risk analysis revealed that CPE colonization was significantly associated with an increased risk of subsequent CPE bacteremia compared with noncolonization (subdistribution hazard ratio [sHR] 13.1, 95% confidence interval [CI] 6.27–27.3, *P* < .001; Figure 2*A*). The estimated probability of CPE bacteremia 30 days after HSCT was significantly higher in CPE-colonized recipients compared with noncolonized recipients (0.20, 95% CI .10–.29 vs .01, 95% CI .004–.02, *P* < .001).

A 1:2 PS matching was performed based on baseline demographics, underlying diseases, type of hematologic malignancies, and HSCT characteristics. After matching, the baseline characteristics between the 2 groups were well-balanced (Table 2; Supplementary Figure 1). After matching, CPE colonization was significantly associated with an increased risk of subsequent CPE bacteremia (17 patients [24%] vs 2 patients [1%], *P* < .001) and subsequent *Enterobacteriaceae* bacteremia (20 patients [29%] vs 9 patients [6%], *P* < .001). This association remained consistent in competing-risk analysis (sHR 19.1, 95% CI 4.42–82.20, *P* < .001; Figure 2*B*). The findings were further corroborated in a sensitivity analysis using unmatched cohorts, where CPE colonization was independently associated with an increased risk of CPE bacteremia (adjusted hazard ratio 11.71, 95% CI 5.55–24.71, *P* < .001; Table 3).

**Table 2.** Baseline Characteristics and Outcome Variables of the Study Population based on the Colonization Status, After Propensity-Score Matching

Variable	Colonized With CPE (N = 70)	Not Colonized With CPE (N = 140)	Total (N = 210)	*P*-Value
Age (y), mean ± SD	49.3 ± 14.1	48.3 ± 14.1	49.0 ± 14.1	.81
Male	45 (64)	91 (65)	136 (65)	>.99
Underlying disease/condition				
Hypertension	20 (29)	39 (28)	59 (28)	>.99
Diabetes mellitus	11 (16)	23 (16)	34 (16)	>.99
Chronic kidney disease	4 (6)	7 (5)	11 (5)	>.99
Liver cirrhosis	0	0	0	>.99
Solid organ transplant	1 (1)	2 (1)	3 (1)	>.99
Solid cancer	1 (1)	4 (3)	5 (2)	.87
Rheumatologic disease	4 (6)	6 (4)	10 (5)	.91
Charlson's comorbidity index	3.4 ± 1.6	3.3 ± 1.4	3.3 ± 1.5	.69
Underlying malignancy				>.99
AML	31 (44)	66 (47)	97 (46)	
ALL	23 (33)	42 (30)	65 (31)	
Multiple myeloma	6 (9)	11 (8)	17 (8)	
MDS	2 (3)	5 (4)	7 (3)	
CML	1 (1)	3 (2)	4 (2)	
Non-Hodgkin's lymphoma	5 (7)	10 (7)	15 (7)	
Hodgkin's lymphoma	1 (1)	2 (1)	3 (1)	
Others	1 (1)	1 (1)	2 (1)	
Type of transplant				>.99
Allogeneic	61 (87)	121 (86)	182 (87)	
Autologous	9 (13)	19 (14)	28 (13)	
Source of donor				.72
Matched, related	14 (20)	21 (15)	35 (17)	
Matched, unrelated	24 (34)	42 (30)	66 (31)	
Mismatched, related	22 (31)	54 (39)	76 (36)	
Mismatched, unrelated	1 (1)	4 (3)	5 (2)	
Conditioning regimen				.54
Myeloablative	70 (100)	137 (98)	207 (99)	
Reduced-intensity or nonmyeloablative	0	3 (2)	3 (1)	
Prior hospitalization	66 (94)	119 (85)	185 (88)	.08
Prior CPE infection	2 (3)^a^	1 (1)^b^	3 (1)	.54
Antibiotics exposure	69 (99)	129 (92)	198 (94)	.12
Gram-negative coverage	69 (99)	128 (91)	197 (94)	.09
Quinolone prophylaxis	15 (21)	33 (24)	48 (23)	.86
Non-*Enterobacteriaceae* bacteremia	11 (16)	16 (11)	27 (12)	.51
Duration from perianal swab to transplantation, mean days ± SD^c^	8.0 ± 6.4	7.5 ± 1.7	7.6 ± 3.9	.25
Duration to neutrophil engraftment, mean days ± SD^d^	12.3 ± 9.0	10.2 ± 6.8	10.9 ± 7.7	.13
30-d mortality after HSCT	1 (1)	2 (1)	3 (1)	.99
90-d mortality after HSCT	10 (14)	5 (4)	15 (7)	.01

Data are presented as number (%) unless otherwise indicated.

Abbreviations: CPE, carbapenemase-producing *Enterobacteriaceae*; SD, standard deviation; AML, acute myeloid leukemia; ALL, acute lymphoblastic leukemia; MDS, myelodysplastic syndrome; CML, chronic myeloid leukemia.

^a^Two patients with CPE bacteremia.

^b^Two patients with CPE bacteremia.

^c^Days from surveillance rectal swab to HSCT.

^d^Neutrophil engraftment was defined as days from cell infusion to the resolution of neutropenia (3 consecutive days with a neutrophil count of ≥500 cells/mm^3^).

**Table 3.** Multivariate Cox Proportional Hazard Model

Characteristic	Univariate Analysis			Multivariate Analysis		
	Hazard ratio	95% Confidence Interval	*P-*Value	Adjusted Hazard Ratio	95% Confidence Interval	*P-*Value
Age	1.01	.98–1.04	.65	…	…	…
Male sex	1.31	.62–2.77	.49	…	…	…
Charlson's comorbidity index	1.22	.98–1.51	.07	1.28	1.03–1.58	.03
Diabetes mellitus	1.11	.42–2.91	.83	…	…	…
Hypertension	1.11	.45–2.73	.82	…	…	…
Solid cancer	2.70	.64–11.37	.18	…	…	…
Chronic kidney disease	2.11	.51–9.05	.30	…	…	…
Liver cirrhosis	0.00	0–Inf	>.99	…	…	…
COPD	0.00	0–Inf	>.99	…	…	…
Rheumatologic diseases	4.44	1.35–14.69	.01	…	…	…
Allogeneic HSCT	4.28	1.02–18.01	.047	…	…	…
Type of hematologic malignancy						
ALL	Reference	…	…	…	…	…
AML	0.26	.10–.70	.007	…	…	…
CML	0.95	.12–7.37	.96	…	…	…
Hodgkin's lymphoma	…	.00–Inf	>.99	…	…	…
Non-Hodgkin's lymphoma	0.16	.02–1.26	.08	…	…	…
MDS	0.51	.19–1.37	.18	…	…	…
MM	0.15	.03–.70	.02	…	…	
Others	0.53	.12–2.39	.41	…	…	…
Solid organ transplant						
Kidney transplantation	8.22	1.12–60.44	.04	…	…	…
Liver transplantation	0.00	.00–Inf	>.99	…	…	…
Quinolone prophylaxis	0.18	.05–.60	.005	0.24	.07–.80	.02
Duration from perianal swab to transplantation	0.96	.84–1.10	.55	…	…	…
CPE colonization	13.09	6.25–27.42	<.001	11.71	5.55–24.71	<.001

Schoenfeld residual shows that the multivariate model did not violate the proportional hazard assumption (*P* = .77).

Abbreviations: COPD, chronic obstructive pulmonary disease; Inf, infinity; HSCT, hematopoietic stem cell transplantation; ALL, acute lymphoblastic leukemia; AML, acute myeloid leukemia; CML, chronic myeloid leukemia; MDS, myelodysplastic syndrome; MM, multiple myeloma; CPE, carbapenemase-producing *Enterobacteriacae*.

The characteristics of CPE bacteremia and non-CPE bacteremia are summarized in Supplementary Table 2. Compared with patients with non-CPE bacteremia, those with CPE bacteremia were less likely to have received quinolone prophylaxis (3/29 [10%] vs 19/47 [40%], *P* < .001) and exhibited a significantly higher 30-day mortality rate after bacteremia (13/29 [45%] vs 4/47 [9%], *P* = .001; Supplementary Table 2). This finding was consistent in the Kaplan–Meier curve (*P* < .001; Supplementary Figure 2) and multivariable Cox's proportional hazard model (adjusted hazard ratio 6.33, 95% CI 2.02–19.85, *P* = .002; Supplementary Table 3). However, there were no significant differences in the proportion of preengraftment infections (17/29 [59%] vs 27/47 [57%], *P* > .99) or suspected infection focus (*P* = .11).

### Microbiological Characteristics of Isolated Carbapenemase-Producing *Enterobacteriaceae*

Seventy CPE isolates were identified from perianal swabs, and 29 CPE isolates were recovered from blood samples (Supplementary Table 4). All CPE isolates recovered from rectal swabs or blood cultures belonged to the *Enterobacteriaceae* family; none were non-*Enterobacteriaceae Enterobacterales*. Among the perianal swab isolates, *K. pneumoniae* (N = 42) was the most common pathogen, followed by *Escherichia coli* (N = 20), *Klebsiella oxytoca* (N = 3), *Citrobacter freundii* (N = 3), *Enterobacter cloacae* (N = 2), *Klebsiella variicola* (N = 2), *Klebsiella aerogenes* (N = 1), *Citrobacter farmeri* (N = 1), and *Leclercia adecarboxylata* (N = 1). In 4 patients, the Xpert Carba-R assay detected carbapenemase genes; however, no pathogens were isolated in CPE cultures. The most common type of carbapenemase was KPC (N = 41), followed by NDM (N = 30), VIM (N = 3), and OXA-48-like (N = 1). Among these, KPC and NDM were most strongly associated with subsequent CPE bacteremia (*P* = .031; Supplementary Figure 3).

Among the 29 CPE isolates recovered from blood samples, the most common pathogen was *K. pneumoniae* (N = 25), followed by *E. coli* (N = 2), *K. oxytoca* (N = 1), and *C. freundii* (N = 1). The distribution of carbapenemase was similar to that of isolates from perianal swab. The predominant carbapenemase type was KPC (N = 22), followed by NDM (N = 6) and OXA-48-like (N = 1). All CPE isolates recovered from the blood of previously CPE-colonized patients were of the same species and exhibited the same carbapenemase type as the strains identified in their earlier perianal swab cultures.

The antibiotic susceptibility patterns of the 29 CPE blood isolates are summarized in Supplementary Table 5. Most isolates were nonresistant to amikacin (28/29 [97%]). However, excluding ceftazidime–avibactam—tested for susceptibility in only 5 isolates—none of the remaining antibiotics exhibited a susceptibility rate of ≥50% among the isolates.

## DISCUSSION

The present study demonstrated that CPE colonization before HSCT was significantly associated with the development of subsequent CPE bacteremia within 100 days after HSCT. This association remained consistent after PS matching and sensitivity analyses. Furthermore, CPE colonization was associated with increased 90-day mortality after transplantation, and CPE bacteremia was significantly associated with higher 30-day mortality after the bacteremia onset compared with non-CPE bacteremia. These findings align with previous studies and underscore the importance of preventing infections caused by pathogens with limited therapeutic options, such as CPE [11–13].

Current guidelines from the IDSA and ECIL suggest that empirical treatment targeting CPE can be considered for patients with hematological malignancies who are colonized with CPE and develop neutropenic fever [2, 5]. However, the evidence supporting this recommendation remains limited. Since the publication of these guidelines, several studies have reported an increased risk of CPE bacteremia in patients with hematologic malignancies colonized with CPE [6, 7, 9, 14–21]. Despite these findings, existing studies are constrained by several limitations, including small sample sizes, uncertainty regarding the timing of CPE screening, and inadequate adjustment for confounding factors [10]. In the present study, these limitations were addressed by analyzing a relatively large cohort and mitigating potential bias from early mortality using PS matching and competing risk analysis.

In our previous study, it was observed that patients with CPE bacteremia were more likely to be colonized with KPC-producing strains compared with those with non-CPE bacteremia [22]. Similar findings have been reported in other studies from East Asia; however, these observations have not been consistently replicated in studies conducted in other regions [11, 20]. It is hypothesized that this discrepancy might be attributed to several factors: presence of hypervirulent *K. pneumoniae* strains in East Asia, presence of additional genes carried on the predominant KPC plasmids in Korea that enhance bloodstream infection fitness, and limited availability of effective therapeutic options for KPC-producing pathogens (eg, ceftazidime–avibactam) [22]. As supporting evidence, our unpublished sequence typing analysis revealed that the majority of CPE-producing *K. pneumoniae* isolates obtained from the hospital environment and patients at Asan Medical Center were classified as ST317 (35/36, [97%]), while a smaller proportion belonged to ST11 (1/36, [3%]), which are known to harbor multiple factors that enhance virulence in bloodstream infections [23–25].

In the present study, most CPE isolates (28/29, [97%]) retrieved from blood were nonresistant to amikacin. Among the isolates tested for ceftazidime–avibactam susceptibility (N = 5), all demonstrated susceptibility. Previous studies have indicated that delayed initiation of appropriate antibiotic therapy in patients who develop bacteremia after HSCT is associated with increased mortality [6, 9]. Therefore, our finding indicating that patients colonized with CPE are at a higher risk of developing CPE bacteremia underscores the potential benefit of empirical therapy targeting CPE in similar clinical settings. Additionally, our results emphasize the importance of routine CPE screening, not only as a critical measure for infection control but also as a reliable predictor of bacteremia characteristics in this patient population.

Our study has several limitations. First, despite the use of PS matching to minimize bias, the retrospective nature of the study might still allow unmeasured confounders to influence our conclusions. Second, as observed in previous East Asian studies where KPC colonization is often identified as a risk factor for CPE, our findings are based on data from a single-center analysis, which limits their generalizability to broader populations. Third, we analyzed only the first episode of *Enterobacteriaceae* bacteremia for simplicity of analysis. Although concerns exist regarding the acquisition of antibiotic resistance in subsequent episodes, most bacteremic patients in our study experienced only one episode of *Enterobacteriaceae* bacteremia during the 100 days following HSCT. Therefore, limiting the analysis to the first episode is unlikely to have significantly impacted our conclusions. Fourth, we were unable to perform susceptibility testing for noncarbapenem antibiotics on CPE isolates colonizing the rectum. This limitation precluded further analysis of the potential impact of other confounding factors, such as quinolone prophylaxis.

In conclusion, pretransplant CPE colonization in HSCT recipients is a risk factor for CPE bacteremia within 100 days posttransplantation. This finding supports the empirical use of CPE-targeted therapy in cases of *Enterobacteriaceae* bacteremia among individuals undergoing HSCT. Additionally, our findings provide evidence for the routine implementation of CPE screening in individuals undergoing HSCT to guide treatment decisions in the posttransplantation period.

## Supplementary Material

ofaf516_Supplementary_Data

## Supplementary Data

Supplementary materials are available at *Open Forum Infectious Diseases* online. Consisting of data provided by the authors to benefit the reader, the posted materials are not copyedited and are the sole responsibility of the authors, so questions or comments should be addressed to the corresponding author.
