# Supplementary material for: Carbapenemase-Producing Enterobacteriaceae Colonization and the Risk of Carbapenemase-Producing Enterobacteriaceae Bacteremia in Hematopoietic Stem Cell Transplant Recipients
Source: Open Forum Infect Dis. 2025 Sep 2;12(9):ofaf516. doi: 10.1093/ofid/ofaf516 (PMC12418090; doi:10.1093/ofid/ofaf516)

**Supplementary Table 1.** The testing, prophylaxis, and empiric treatment protocol during HSCT of Asan Medical Center

| **Protocol** | **Note** |
| --- | --- |
| **Pre-HSCT pathogen-specific testing** |  |
| TB interferon gamma test | Isoniazid for 9 months |
| Serum galactomannan | consult to ID physicians |
| HBsAg, IgG anti-HBc, IgM anti-HBc | Entecavir for 6 to 12 month post-transplant |
| Anti-HCV | Treatment against HCV |
| HIV Ag/Ab | Treatment against HIV |
| CMV Ab IgG, IgM and DNA | Letemovir prophylaxis to 3 month post-transplant |
| HSV Ab IgG and IgM | Acyclovir prophylaxis to 12 month post-transplant |
| Varicella zoster virus IgG and IgM | Acyclovir prophylaxis to 12 month post-transplant |
| EBV-NA IgG, EBV-VCA IgG, IgM and DNA |  |
| Toxoplasma Ab IgG and IgM | Trimethoprim/sulfamethoxazole from engraftment to 6 months |
| Syphilis regain (RPR) | Treatment against syphilis |
| Rectal swab for CPE screening | At time of admission |
| **Prophylactic antibiotics** |  |
| Acyclovir | HSV or VZV antibody positive |
| Micafungin | Given from day 0 to resolution of neutropenia |
| Posaconazole | High-risk patients of invasive mold infection;  GVHD |
| Trimethoprim-sulfamethoxazole | Universal prophylaxis; administer from engraftment through 6 months |
| Ciprofloxacin | Given from day 0 to resolution of neutropenia; withheld it when patients are already receiving broad-spectrum antibiotics; not recommended after 2023 due to concern for breakthrough infection due to resistant organism |
| **Emprical antibiotics/antifungal for neutropenic fever** | |
| Cefazolin and ceftazidime | Initial empirical treatment for neutropenic fever |
| Cefepime or piperacillin/tazobactam | Persistent fever lasting more than 3 days despite empirical antibiotic therapy |
| Ambisome or caspofungin | Persistent fever lasting more than 3 days and combined hemodynamic instability, or persistent fever lasting more than 5 days |
| Meropenem | Persistent fever lasting more than 3 days and combined hemodynamic instability |
| Amikacin | Persistent fever lasting more than 3 days and combined hemodynamic instability |
| Vancomycin or teicoplanin | Central venous catheter infection, hemodynamic instability, bacteremia due to gram positive pathogen, combined soft tissue or skin infection |
| Ceftazidime/avibactam | Fever with hemodynamic instability in CPE colonizer; De-escalate to piperacillin/tazobactam or cefepime if CPE bacteremia is ruled out within 2 days |

**Supplementary Table 2.** Baseline characteristics of patients with CPE bacteremia and non-CPE bacteremia.

| **Variable** | **CPE bacteremia**  (N=29) | **Non-CPE bacteremia**  (N=47) | **Total**  (N=76) | **p*-*value** |
| --- | --- | --- | --- | --- |
| **Age (years), mean** ± **SD** | 53.2±13.6 | 51.1±15.1 | 51.9±14.5 | 0.54 |
| **Male** | 18 (62) | 30 (64) | 48 (63) | >0.99 |
| **Underlying disease/condition** |  |  |  |  |
| Hypertension | 6 (21) | 7 (15) | 13 (17) | 0.74 |
| Diabetes mellitus | 5 (17) | 7 (15) | 12 (16) | >0.99 |
| Chronic kidney disease | 2 (7) | 2 (4) | 4 (5) | >0.99 |
| Liver cirrhosis | 0 | 1 (2) | 1 (1) | >0.99 |
| Solid organ transplant | 1 (3) | 0 | 1 (1) | 0.81 |
| Solid cancer | 2 (7) | 0 | 2 (3) | 0.28 |
| Rheumatologic disease | 3 (10) | 1 (2) | 4 (5) | 0.30 |
| Charlson’s comorbidity index | 4.0±1.9 | 3.5±1.4 | 3.7±1.6 | 0.22 |
| **Underlying malignancy** |  |  |  | 0.03 |
| AML | 6 (21) | 14 (30) | 20 (26) |  |
| ALL | 11 (38) | 3 (6) | 14 (18) |  |
| Multiple myeloma | 2 (7) | 5 (11) | 7 (9) |  |
| MDS | 6 (21) | 16 (34) | 22 (29) |  |
| CML | 1 (3) | 1 (2) | 2 (3) |  |
| Non-Hodgkin’s lymphoma | 1 (3) | 6 (13) | 7 (9) |  |
| Hodgkin’s lymphoma | 0 | 0 | 0 |  |
| Others | 2 (7) | 2 (4) | 4 (5) |  |
| **Type of transplant** |  |  |  | 0.50 |
| Allogeneic | 27 (93) | 40 (85) | 67 (88) |  |
| Autologous | 2 (7) | 7 (15) | 9 (12) |  |
| **Quinolone prophylaxis** | 3 (10) | 19 (40) | 22 (29) | 0.01 |
| **CPE colonization** | 17 (59) | 3 (6) | 20 (26) | <0.001 |
| **Duration to neutrophil engraftment, mean days** ± **SD** | 13.2±8.9 | 14.8±13.3 | 14.3±11.9 | 0.92 |
| **Pre-engraftment bacteremia** | 17(59) | 27(57) | 44 (58) | >0.99 |
| **Suspected infection focus** |  |  |  | 0.11 |
| Primary | 20 (69) | 41 (87) | 61 (80) |  |
| CRBSI | 2 (7) | 3 (6) | 5 (7) |  |
| Intraabdominal | 6 (21) | 2 (4) | 8 (11) |  |
| Pneumonia | 1 (3) | 0 | 1 (1) |  |
| Musculoskeletal | 0 | 1 (2) | 1 (1) |  |
| **Neutrophil count (cells/mm^3^),  mean ± SD** | 569.1±1310.7 | 627.5±2704.9 | 605.2±2264.8 | 0.90 |
| **Duration of neutropenia  before bacteremia,  mean days ± SD** | 21.7±42.8 | 14.4±27.9 | 17.2±34.3 | 0.42 |
| **30-day mortality after bacteremia** | 13 (45) | 4 (9) | 17 (22) | 0.001 |

Note. Data are presented as number (%) unless otherwise indicated.
CPE, carbapenemase-producing *Enterobacteriaceae*; SD, standard deviation; AML, acute myeloid leukemia; ALL, acute lymphoblastic leukemia; MDS, myelodysplastic syndrome; CML, chronic myeloid leukemia; CRBSI, catheter-related bloodstream infection.

**Supplementary Table 3.** Multivariate Cox proportional hazard model including CPE bacteremia**.**

| Characteristic | Univariate analysis | | | Multivariate analysis | | | |
| --- | --- | --- | --- | --- | --- | --- | --- |
|  | Hazard ratio | 95% Confidence interval | p-value | | Adjusted hazard ratio | 95% Confidence interval | p- value |
| Age | 1.01 | 0.98–1.04 | 0.60 | |  |  |  |
| Male sex | 3.05 | 0.88 – 10.62 | 0.08 | | 6.35 | 1.54 – 26.23 | 0.01 |
| Charlson’s comorbidity index | 1.17 | 0.88 -1.55 | 0.28 | | 1.34 | 1.00 – 1.80 | 0.048 |
| Diabetes mellitus | 0.67 | 0.15 – 2.95 | 0.60 | | 0.25 | 0.04 – 1.49 | 0.13 |
| Hypertension | 1.56 | 0.51 – 4.79 | 0.44 | |  |  |  |
| Solid cancer | 15.54 | 3.10 – 77.95 | 0.001 | |  |  |  |
| Chronic kidney disease | 1.15 | 0.15 – 8.65 | 0.89 | |  |  |  |
| Liver cirrhosis | 0.00 | 0 – Inf | >0.99 | |  |  |  |
| Rheumatologic diseases | 4.96 | 1.41 – 17.41 | 0.01 | | 4.87 | 1.06 – 22.52 | 0.04 |
| Autogenic HSCT | 0.00 | 0 – Inf | >0.99 | |  |  |  |
| Type of hematologic malignancy |  |  |  | |  |  |  |
| ALL | Reference |  |  | |  |  |  |
| AML | 0.59 | 0.16 – 2.19 | 0.43 | |  |  |  |
| CML | 2.08 | 0.24 – 17.91 | 0.50 | |  |  |  |
| Non-Hodgkin’s lymphoma | 0.38 | 0.04 – 3.29 | 0.38 | |  |  |  |
| MDS | 0.52 | 0.14 – 1.93 | 0.33 | |  |  |  |
| MM | 0.00 | 0 – Inf | >0.99 | |  |  |  |
| Others | 1.98 | 0.38 – 10.25 | 0.41 | |  |  |  |
| Solid organ transplant |  |  |  | |  |  |  |
| Kidney transplantation | 0.00 | 0 – Inf | >0.99 | |  |  |  |
| Quinolone prophylaxis | 0.70 | 0.23 – 2.16 | 0.54 | |  |  |  |
| CPE bacteremia | 6.60 | 2.15 – 20.28 | 0.001 | | 6.33 | 2.02 – 19.85 | 0.002 |

Schoenfeld residual shows that the multivariate model did not violate the proportional hazard assumption (p=0.79).

COPD, chronic obstructive pulmonary disease; Inf, infinity; HSCT, hematopoietic stem cell transplantation; ALL, acute lymphoblastic leukemia; AML, acute myeloid leukemia; CML, chronic myeloid leukemia; MDS, myelodysplastic syndrome; MM, multiple myeloma; CPE, carbapenemase-producing *Enterobacteriaceae.*

**Supplementary Table 4.** Characteristics of 79 colonized CPE species and 29 CPE blood isolates.

| **Organism type** | **Colonized (N=79)** | **Blood isolates (N=29)** |
| --- | --- | --- |
| **Species** |  |  |
| *Klebsiella pneumoniae* | 42 | 25 |
| *Escherichia coli* | 20 | 2 |
| *Klebsiella oxytoca* | 3 | 1 |
| *Cirobacter freundii* | 3 | 1 |
| *Enterobacter cloacae* | 2 |  |
| *Klebsiella variicola* | 2 |  |
| *Klebsiella aerogenes* | 1 |  |
| *Citrobacter farmeri* | 1 |  |
| *Leclercia adecarboxylata* | 1 |  |
| PCR-only^a^ | 4 |  |
| Multiple pathogen^b^ | 9 |  |
| **Carbapenemase** |  |  |
| KPC | 41 | 22 |
| NDM | 30 | 6 |
| OXA-48-like | 1 | 1 |
| VIM | 3 |  |
| Multiple carbapenemase ^c^ | 5 |  |

CPE, carbapenemase-producing *Enterobacteriaceae*; PCR, polymerase chain reaction; KPC, *Klebsiella*-producing carbapenemase; NDM, New Delhi metallo-beta-lactamase; OXA-48, oxacillinase-48-like beta-lactamase carbapenemase; VIM, Verona integrin-encoded metallo-beta-lactamase. ^a^ one NDM, one KPC, one VIM, and one OXA-48.
^b^ Six patients with *Klebsiella pneumoniae* and *Escherichia coli*, one patient with *Escherichia coli* and *Klebsiella oxytoca*, one patient with *Escherichia coli* and *Cirobacter freundii*, and one patient with *Cirobacter freundii* and *Klebsiella oxytoca*.
^c^ Three patients with KPC and NDM and two patients with NDM and VIM.

**Supplementary table 5**. Antibiotics susceptibility of 29 CPE blood isolates.

| Pathogen | Carbapenemase | SAM | AMK | AMP | AZT | CAZ | CTX | CRO | CAZ/AVI | FOX | CFZ | CST | CIP | FEP | CFP | GEN | IMI | LVX | MEM | PIP/TAZ | TMP/SMX | TET | TGC | TOR |
| --- | --- | --- | --- | --- | --- | --- | --- | --- | --- | --- | --- | --- | --- | --- | --- | --- | --- | --- | --- | --- | --- | --- | --- | --- |
| *K. pneumoniae* | KPC | > 16/8 | ≤ 16 | >16 | >16 | >16 | >32 |  |  | >16 | >4 | ≤2 | >2 | >16 | >16 | >8 | >8 | >4 | >8 | >64 | >2/38 | >8 |  | >8 |
| *K. oxytoca* | NDM | > 16/8 | ≤ 16 | >16 | ≤4 | >16 | 32 |  |  | >16 | >4 | ≤2 | ≤1 | 8 | >16 | ≤4 | 8 | ≤2 | 8 | 64 | ≤2/38 | ≤4 |  | ≤4 |
| *K. pneumoniae* | NDM | > 16/8 | ≤ 16 | >16 | >16 | >16 | >32 |  |  | >16 | >4 | ≤2 | >2 | >16 | >16 | >8 | >8 | >4 | >8 | >64 | >2/38 | >8 |  | >8 |
| *K. pneumoniae* | KPC | > 16/8 | ≤ 16 | >16 | >16 | >16 | >32 |  |  | >16 | >4 | ≤2 | >2 | >16 | >16 | >8 | 8 | >4 | >8 | >64 | >2/38 | >8 |  | >8 |
| *C. freundii* | NDM | > 16/8 | ≤ 16 | >16 | ≤4 | >16 | >32 |  |  | >16 | >4 | ≤2 | >2 | 16 | >16 | ≤4 | 4 | >4 | 8 | 64 | >2/38 | ≤4 |  | ≤4 |
| *K. pneumoniae* | KPC | > 16/8 | ≤ 16 | >16 | >16 | >16 | >32 |  |  | >16 | >4 | ≤2 | >2 | >16 | >16 | >8 | 8 | >4 | >8 | >64 | >2/38 | ≤4 |  | 8 |
| *K. pneumoniae* | KPC | > 16/8 | ≤ 16 | >16 | >16 | >16 | >32 |  |  | >16 | >4 | ≤2 | >2 | >16 | >16 | >8 | 8 | >4 | >8 | >64 | >2/38 | ≤4 |  | 8 |
| *K. pneumoniae* | KPC | > 16/8 | ≤ 16 | >16 | >16 | >16 | >32 |  |  | >16 | >4 | 4 | >2 | >16 | >16 | ≤4 | >8 | >4 | >8 | >64 | >2/38 | ≤4 |  | ≤4 |
| *K. pneumoniae* | KPC | > 16/8 | ≤ 16 | >16 | >16 | >16 | >32 |  |  | >16 | >4 | ≤2 | >2 | >16 | >16 | ≤4 | 2 | >4 | 2 | >64 | >2/38 | >8 |  | 8 |
| *K. pneumoniae* | KPC | > 16/8 | ≤ 16 | >16 | >16 | >16 | >32 |  |  | >16 | >4 | ≤2 | >2 | >16 | >16 | >8 | >8 | >4 | >8 | >64 | >2/38 | 8 |  | 8 |
| *K. pneumoniae* | NDM | > 16/8 | ≤ 16 | >16 | >16 | >16 | >32 |  |  | >16 | >4 | ≤2 | >2 | >16 | >16 | >8 | >8 | >4 | >8 | >64 | >2/38 | >8 |  | >8 |
| *K. pneumoniae* | KPC | > 16/8 | ≤ 16 | >16 | >16 | >16 | >32 |  |  | >16 | >4 | ≤2 | >2 | >16 | >16 | >8 | >8 | >4 | >8 | >64 | >2/38 | 8 |  | >8 |
| *K. pneumoniae* | KPC | > 16/8 | ≤ 16 | >16 | >16 | >16 | >32 |  |  | >16 | >4 | ≤2 | >2 | >16 | >16 | ≤4 | >8 | >4 | >8 | >64 | >2/38 | >8 |  | >8 |
| *K. pneumoniae* | KPC | > 16/8 | ≤ 16 | >16 | >16 | >16 | >32 |  |  | >16 | >4 | ≤2 | >2 | >16 | >16 | >8 | 8 | >4 | >8 | >64 | >2/38 | >8 |  | >8 |
| *K. pneumoniae* | KPC | > 16/8 | ≤ 16 | >16 | >16 | >16 | >32 |  |  | >16 | >4 | ≤2 | >2 | >16 | >16 | >8 | >8 | >4 | >8 | >64 | >2/38 | >8 |  | >8 |
| *K. pneumoniae* | OXA-48 | > 16/8 | >32 | >16 | >16 | >16 | >32 |  |  | >16 | >4 | ≤2 | >2 | >16 | >16 | >8 | 4 | >4 | >8 | >64 | >2/38 | ≤4 |  | >8 |
| *K. pneumoniae* | KPC | > 16/8 | ≤ 16 | >16 | >16 | >16 | >32 |  |  | >16 | >4 | ≤2 | >2 | >16 | >16 | >8 | >8 | >4 | >8 | >64 | >2/38 | >8 |  | >8 |
| *K. pneumoniae* | KPC | > 16/8 | ≤ 16 | >16 | >16 | >16 | >32 |  |  | >16 | >4 | ≤2 | >2 | >16 | >16 | >8 | 8 | >4 | >8 | >64 | >2/38 | >8 |  | >8 |
| *K. pneumoniae* | KPC | > 16/8 | ≤ 16 | >16 | >16 | >16 | >32 |  |  | >16 | >4 | ≤2 | >2 | >16 | >16 | ≤4 | 8 | >4 | 8 | >64 | >2/38 | >8 |  | ≤4 |
| *K. pneumoniae* | KPC | > 16/8 | ≤ 16 | >16 | >16 | >16 | >32 |  |  | >16 | >4 | ≤2 | >2 | >16 | >16 | ≤4 | >8 | >4 | >8 | >64 | >2/38 | >8 |  | 8 |
| *K. pneumoniae* | NDM | > 16/8 | ≤ 16 | >16 | >16 | >16 | >32 |  |  | >16 | >4 | ≤2 | >2 | >16 | >16 | ≤4 | >8 | ≤2 | >8 | >64 | >2/38 | ≤4 |  | 8 |
| *K. pneumoniae* | KPC | > 16/8 | ≤ 16 | >16 | >16 | 16 | >32 |  |  | >16 | >4 | ≤2 | >2 | ≤2 | >16 | ≤4 | 4 | >4 | >4 | >64 | >2/38 | >8 |  | 8 |
| *K. pneumoniae* | KPC | > 16/8 | ≤ 16 | >16 | >16 | >16 | >32 |  |  | >16 | >4 | ≤2 | >2 | >16 | >16 | ≤4 | 8 | >4 | >4 | >64 | >2/38 | >8 |  | 8 |
| *K. pneumoniae* | KPC | > 16/8 | ≤ 16 | >16 | >16 | 16 | >32 |  |  | >16 | >4 | ≤2 | >2 | ≤2 | >16 | ≤4 | >8 | >4 | >8 | >64 | >2/38 | >8 |  | 8 |
| *K. pneumoniae* | KPC | > 16/8 | 8 | >16 | >16 | >16 |  | >4 | 2/4 | >16 | >16 | ≤1 | >2 | >16 | >16 | ≤2 | >8 | >4 | 16 | >64 | >2/38 |  | >4 |  |
| *E. coli* | NDM | > 16/8 | ≤4 | >16 | >16 | >16 |  | >4 | ≤ 0.25/4 | >16 | >16 | ≤1 | ≤0.125 | >16 | >16 | >8 | >8 | ≤1 | 4 | >64 | >2/38 |  | 1 |  |
| *K. pneumoniae* | KPC | > 16/8 | ≤4 | >16 | >16 | >16 |  | >4 | 2/4 | >16 | >16 | ≤1 | >2 | >16 | >16 | ≤2 | >8 | >4 | 32 | >64 | >2/38 |  | >4 |  |
| *K. pneumoniae* | KPC | > 16/8 | ≤4 | >16 | >16 | >16 |  | >4 | ≤ 0.25/4 | >16 | >16 | ≤1 | >2 | >16 | >16 | ≤2 | 8 | >4 | 8 | >64 | >2/38 |  | 4 |  |
| *E. coli* | KPC | > 16/8 | ≤4 | >16 | >16 | >16 |  | >4 | ≤ 0.25/4 | >16 | >16 | ≤1 | >2 | >16 | >16 | >8 | >8 | >4 | >32 | >64 | >2/38 |  | ≤1 |  |

KPC, Klebsiella-producing carbapenemase; NDM, New Delhi metallo-beta-lactamase; OXA-48, Oxacillinase-48-type carbapenemase; SAM, ampicillin/sulbactam; AMK, amikacin; AMP, ampicillin; AZT, aztreonam; CAZ, ceftazidime; CTX, cefotaxime; CRO, ceftriaxone; CAZ/AVI, ceftazidime/avibactam; FOX, cefoxitin; CFZ, cefazolin; CST, colistin; CIP, ciprofloxacin; FEP, cefepime; CFP, cefoperazone; GEN, gentamicin; IMI, imipenem/cilastatin; LVX, levofloxacin; MEM, meropenem; PIP/TAZ, piperacillin/tazobactam; TMP/SMX, trimethoprim/sulfamethoxazole; TET, tetracycline; TGC, tigecycline; TOR, tobramycin.

Antimicrobial susceptibility testing was performed according to the CLSI M100, 32nd edition.

**Supplementary Figure 1.** Standardized mean difference (SMD) of the unmatched and matched cohorts. Red dots indicate the PS-matched cohort, while empty dots indicate the unmatched cohort. The dashed line represents the acceptable threshold of the SMD.


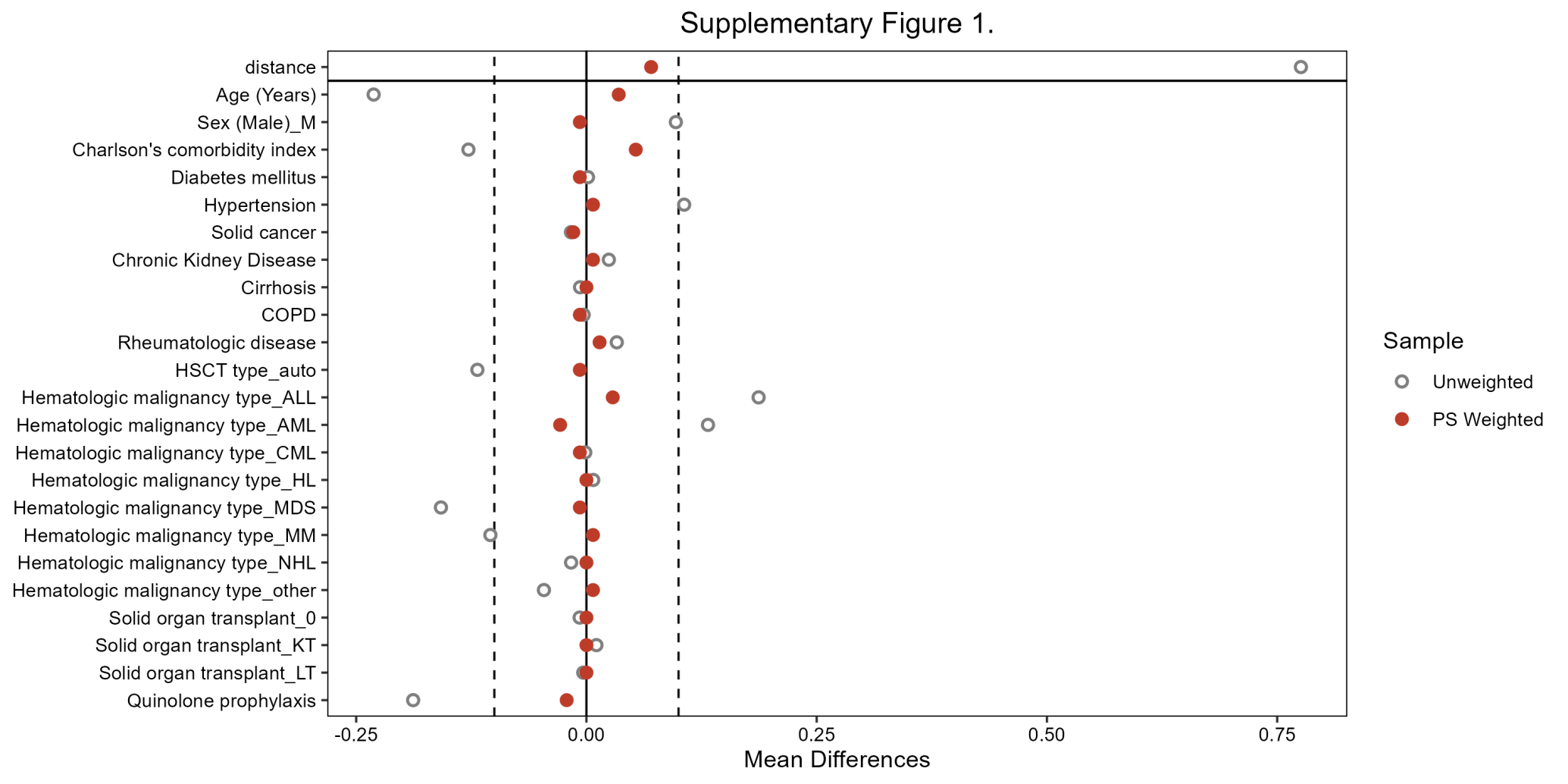


COPD, chronic obstructive pulmonary disease; ALL, acute lymphoblastic leukemia; AML, acute myeloid leukemia; CML, chronic myeloid leukemia; HL, Hodgkin’s lymphoma; MDS, myelodysplastic syndrome; MM, multiple myeloma; NHL, non-Hodgkin’s lymphoma; KT, kidney transplantation; LT, liver transplantation.

**Supplementary Figure 2.** Kaplan-Meier curve for 30-day mortality according to causative pathogen of bacteremia.


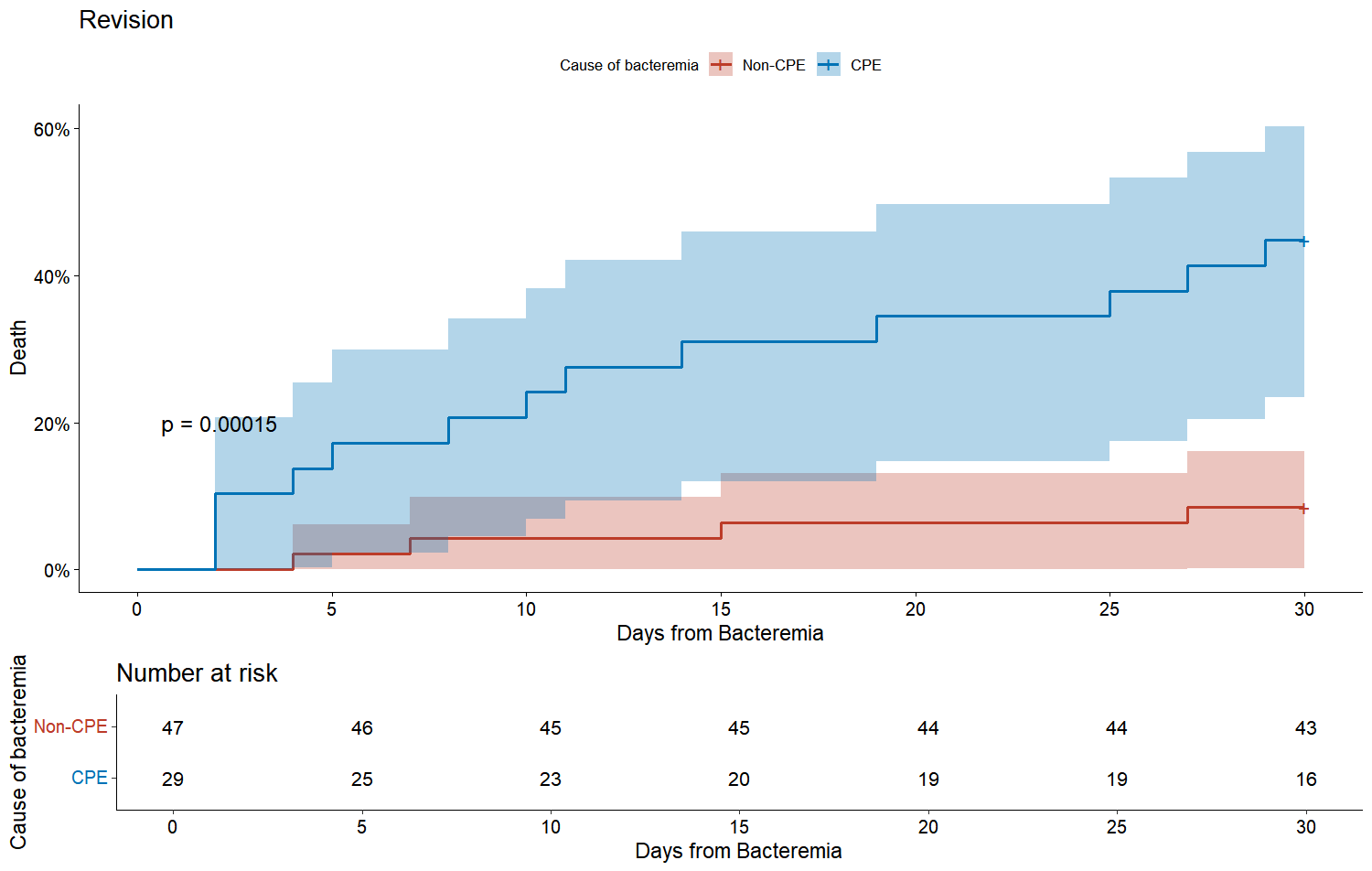


**Supplementary Figure 3.** Kaplan-Meier curve for developing CPE bacteremia according to colonized CPE type.


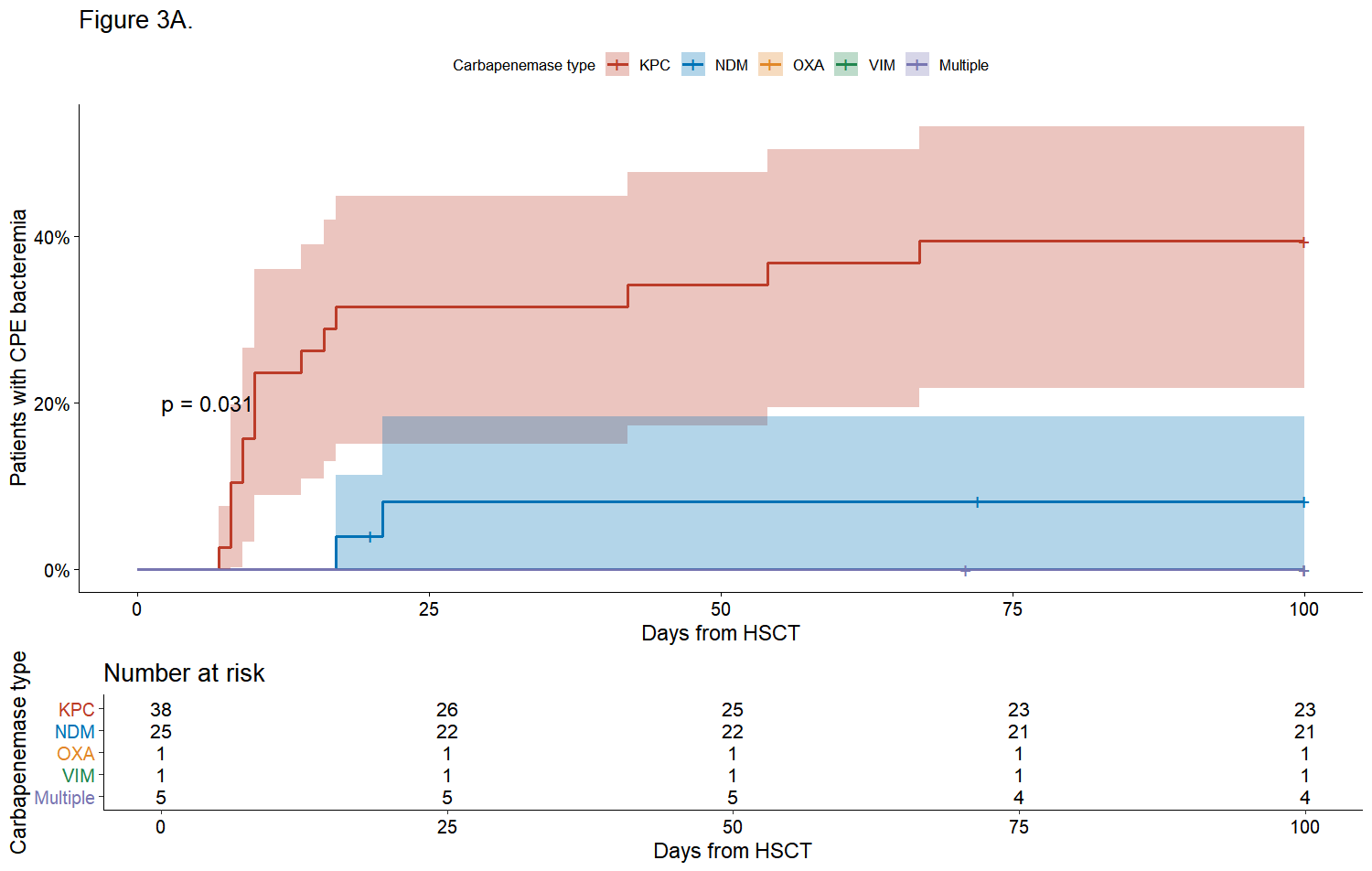

Supplement: ofaf516_Supplementary_Data [file ofaf516_supplementary_data.docx]
